# Radiofrequency Treatment Attenuates Age-Related Changes in Dermal–Epidermal Junctions of Animal Skin

**DOI:** 10.3390/ijms25105178

**Published:** 2024-05-09

**Authors:** Kyung-A Byun, Hyoung Moon Kim, Seyeon Oh, Sosorburam Batsukh, Kuk Hui Son, Kyunghee Byun

**Affiliations:** 1 Department of Anatomy & Cell Biology, College of Medicine, Gachon University, Incheon 21936, Republic of Korea; 2 LIBON Inc., Incheon 22006, Republic of Korea; 3 Functional Cellular Networks Laboratory, Lee Gil Ya Cancer and Diabetes Institute, Gachon University, Incheon 21999, Republic of Korea; 4 Maylin Anti-Aging Center Ilsan, Goyang 10391, Republic of Korea; 5 Department of Thoracic and Cardiovascular Surgery, Gachon University Gil Medical Center, Gachon University, Incheon 21565, Republic of Korea; 6 Department of Health Sciences and Technology, Gachon Advanced Institute for Health & Sciences and Technology (GAIHST), Gachon University, Incheon 21999, Republic of Korea

**Keywords:** aged mice skin, bipolar, dermal–epidermal junction, heat shock protein, monopolar, Piezo1, radiofrequency

## Abstract

The dermal–epidermal junction (DEJ) is essential for maintaining skin structural integrity and regulating cell survival and proliferation. Thus, DEJ rejuvenation is key for skin revitalization, particularly in age-related DEJ deterioration. Radiofrequency (RF) treatment, known for its ability to enhance collagen fiber production through thermal mechanisms and increase heat shock protein (HSP) expression, has emerged as a promising method for skin rejuvenation. Additionally, RF activates Piezo1, an ion channel implicated in macrophage polarization toward an M2 phenotype and enhanced TGF-β production. This study investigated the impact of RF treatment on HSP47 and HSP90 expression, known stimulators of DEJ protein expression. Furthermore, using in vitro and aged animal skin models, we assessed whether RF-induced Piezo1 activation and the subsequent M2 polarization could counter age-related DEJ changes. The RF treatment of H_2_O_2_-induced senescent keratinocytes upregulated the expression of HSP47, HSP90, TGF-β, and DEJ proteins, including collagen XVII. Similarly, the RF treatment of senescent macrophages increased Piezo1 and CD206 (M2 marker) expression. Conditioned media from RF-treated senescent macrophages enhanced the expression of TGF-β and DEJ proteins, such as nidogen and collagen IV, in senescent fibroblasts. In aged animal skin, RF treatment increased the expression of HSP47, HSP90, Piezo1, markers associated with M2 polarization, IL-10, and TGF-β. Additionally, RF treatment enhanced DEJ protein expression. Moreover, RF reduced lamina densa replication, disrupted lesions, promoted hemidesmosome formation, and increased epidermal thickness. Overall, RF treatment effectively enhanced DEJ protein expression and mitigated age-related DEJ structural changes by increasing HSP levels and activating Piezo1.

## 1. Introduction

The dermal–epidermal junction (DEJ), positioned between the epidermis and dermis, consists of the basement membrane (BM) and anchoring proteins [1,2]. The BM, resembling a sheet-like matrix, contains highly organized extracellular matrix (ECM) proteins, including laminin-511 and collagen IV, interconnected by nidogen, proteoglycan, and perlecan [3,4]. Anchoring proteins, such as integrins, laminin-322, collagen VII, and collagen XVII, play pivotal roles in enhancing dermal–epidermal connectivity [5]. While keratinocytes and dermal fibroblasts synthesize these DEJ proteins, collagen XVII is primarily synthesized by keratinocytes, whereas nidogen is mainly synthesized by fibroblasts [6,7,8]. Distinguishing itself from fibrillar collagen, collagen XVII is a transmembrane collagen [9,10].

Basal keratinocytes contain hemidesmosomes, facilitating robust connections between keratinocytes and the BM. Collagen XVII co-localizes with hemidesmosomes and can bind integrin α6, laminin-332, and collagen IV within the lamina densa and lamina lucida of the BM [11,12,13], thereby reinforcing keratinocyte adhesion to the underlying lamina densa and lamina lucida [14]. However, the DEJ undergoes aging-related changes characterized by the diminished expression of laminin-332, integrin β4, collagen IV, collagen VII, and collagen XVII in the skin [15,16,17,18,19,20].

Transforming growth factor beta (TGF-β) plays a pivotal role in the synthesis of various collagen fibers, encompassing types I–V, and BM proteins such as laminin and perlecan [21]. Senescent fibroblasts exhibit diminished TGF-β expression, leading to decreased collagen IV synthesis [20].

Various matrix metalloproteinases (MMPs) are upregulated with aging, contributing to DEJ protein degradation. The MMP-mediated degradation of collagen XVII in aged mouse skin has been reported [22]. Ultraviolet (UV) radiation exposure decreases collagen XVII expression in keratinocytes, accompanied by increased MMP activity [23]. Additionally, reduced collagen XVII expression occurs in chronologically aged skin, photoaged skin, and areas exposed to acute UV radiation [23].

In aged animal skin, reductions in the number of hemidesmosomes and epidermal thickness are evident, correlating with reduced collagen XVII expression [24]. Beyond its structural role in the BM, collagen XVII is also involved in skin rejuvenation by enhancing skin stem cell renewal potential and facilitating structural recovery [24]. Increased collagen XVII expression can attenuate epidermal thinning in aged mice [22,24]. Additionally, peptides that enhance BM components such as laminins and collagen XVII have shown promise in reducing skin wrinkles [25].

Radiofrequency (RF) and ultrasound (US) technologies are frequently employed to enhance collagen synthesis through thermal effects [26,27]. These modalities also upregulate collagen XVII and glycosaminoglycan expression, accompanied by elevated levels of heat shock protein 47 (HSP47) [28]. This suggests a coordinated mechanism wherein HSP47, known to be upregulated in response to heat stimulation, plays a crucial role in increasing collagen synthesis as a collagen-binding glycoprotein [29,30]. Additionally, HSP90, also upregulated in response to heat shock, promotes collagen synthesis by enhancing TGF-β-induced SMAD2/3 phosphorylation [31].

The Piezo1 channel in the cellular membrane induces Ca^2+^ influx upon opening in response to various mechanical stimuli, including matrix stiffness, shear stress, radial pressure, membrane stretching, and compression [32]. Both US and shockwave therapies activate Piezo1 [33,34,35]. Activated Piezo1 participates in diverse signaling pathways in inflammation, cell proliferation, and fibrosis [36]. Furthermore, Piezo1 promotes M2 macrophage polarization [37], a process essential for anti-inflammatory responses and tissue repair [38,39,40]. Macrophages can be classified into two main phenotypes: M1, characterized by pro-inflammatory properties, and M2, characterized by anti-inflammatory characteristics [38,39]. M2 macrophages secrete TGF-β and interleukin 10 (IL-10), which promote ECM production [40].

Previous studies have demonstrated that RF can increase nuclear factor erythroid 2-related factor 2 (NRF2) expression, thereby enhancing macrophage polarization toward the M2 phenotype and elevating IL-10 levels [41]. Elevated IL-10 levels are associated with the reduced production of advanced glycated end products and the downregulation of its receptor (RAGE), along with decreased nuclear factor-kappa B (NF-κB) expression in aged mice [41]. These changes induced by RF radiation significantly contribute to collagen fiber accumulation in aged skin [41].

Additionally, combination therapy involving both RF and US has shown promise in increasing collagen XVII expression [28]. However, it remains uncertain whether RF alone can activate Piezo1, a potential mechanism for increasing DEJ proteins. Thus, we hypothesized that RF can enhance DEJ protein expression by upregulating HSPs and Piezo1, thereby facilitating skin rejuvenation. Specifically, RF-induced Piezo1 activation may stimulate M2 macrophage polarization, resulting in heightened IL-10 secretion. Elevated IL-10 levels, in turn, promote TGF-β production, thus enhancing nidogen and collagen IV expression in fibroblasts. Additionally, RF augments HSP47 and HSP90 expression, further boosting TGF-β levels in keratinocytes. Increased TGF-β levels promote collagen XVII expression and mitigate DEJ structural changes, ultimately promoting skin rejuvenation. These hypotheses were rigorously evaluated using both in vitro and in vivo animal models.

In the bipolar RF mode, an electrical current flow between two electrodes, while in the monopolar mode, it flows from one electrode to a grounding pad [42]. Notably, our previous study revealed a more significant increase in M2 polarization and IL-10 levels when the monopolar mode preceded the bipolar mode radiation compared to other sequences; however, the precise mechanism remains elusive [41]. Additionally, we hypothesized that various combinations and sequences of monopolar and bipolar RF modes (10:0, 5:5, 2:8, and 0:10) could also impact M2 polarization in an animal model.

Understanding the mechanisms of skin aging and rejuvenation is crucial for effective therapeutic strategies. Our study examined the role of RF-induced Piezo1 activation in DEJ protein expression, advancing non-invasive skin rejuvenation. Ultimately, our findings may lead to innovative skincare interventions for healthy, youthful skin aging.

## 2. Results

### 2.1. RF Treatment Increases HSP47, HSP90, TGF-β, and DEJ Protein Levels in Senescent Keratinocytes

**Figure 1:**
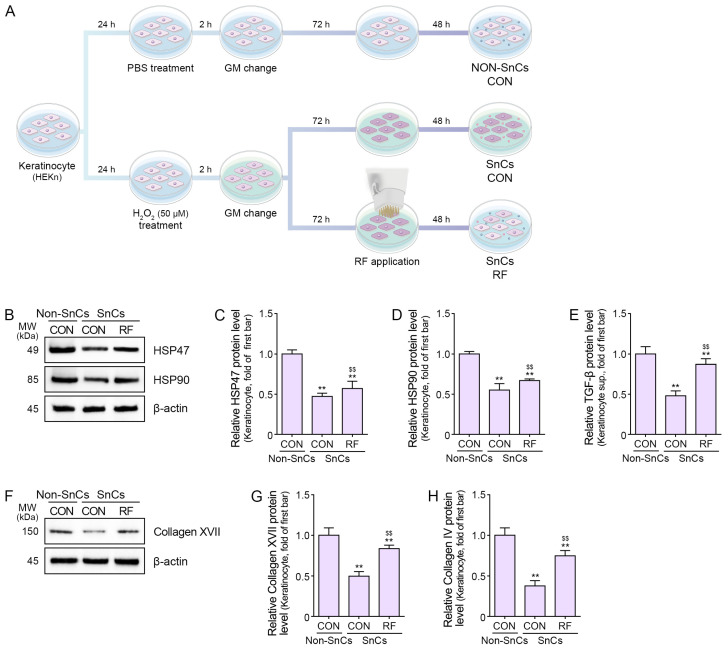
Expression of HSP47, HSP90, TGF-β, and DEJ proteins following RF treatment in senescent keratinocytes. (**A**) Schematic representation of non-senescent keratinocytes, senescent keratinocytes, and RF treatments administered to senescent keratinocytes. (**B**–**H**) Proteins were extracted from senescent keratinocytes following RF treatment and subjected to the following assays. (**B**–**D**) Western blot analysis of HSP47 and HSP90. (**E**) ELISA results for TGF-β. (**F**,**G**) Western blot analysis of collagen XVII. (**H**) ELISA results for collagen IV. Data are presented as mean ± SD of three independent experiments. **, *p* < 0.01 vs. first bar; $$, *p* < 0.01 vs. second bar (Mann–Whitney U test). CON, control; DEJ, dermal–epidermal junction; ELISA, enzyme-linked immunosorbent assay; GM, growth medium; HSP47, heat shock protein 47; HSP90, heat shock protein 90; MW, molecular weight; PBS, phosphate-buffered saline; RF, radiofrequency; SD, standard deviation; SnCs, senescent; TGF-β, transforming growth factor beta.

To establish a senescent keratinocyte model, we induced senescence using H_2_O_2_ treatment followed by RF radiation (Figure 1A). The expression of p21 and p16 which are frequently used senescence markers [43] were evaluated to establish whether H_2_O_2_ treatment induced proper cellular senescence. Both *P21* and *P16* were increased by H_2_O_2_ treatment (Figure S1). The expression levels of HSP47, HSP90, and TGF-β were decreased in the H_2_O_2_-induced senescence keratinocyte. RF treatment significantly increased the protein levels of HSP47 and HSP90 in senescent keratinocytes (Figure 1B–D), along with higher levels of TGF-β expression (Figure 1E). The expression levels of collagen XVII and collagen IV were decreased in the H_2_O_2_-induced senescence keratinocyte. RF treatment also induced the elevated expression of collagen XVII and collagen IV in senescent keratinocytes (Figure 1F–H).

### 2.2. RF Treatment Increases M2 Polarization, as Well as Piezo1 and IL-10 Expression, in Senescent Macrophages and Enhances TGF-β, SMAD2/3, and DEJ Protein Expression in Senescent Fibroblasts

**Figure 2:**
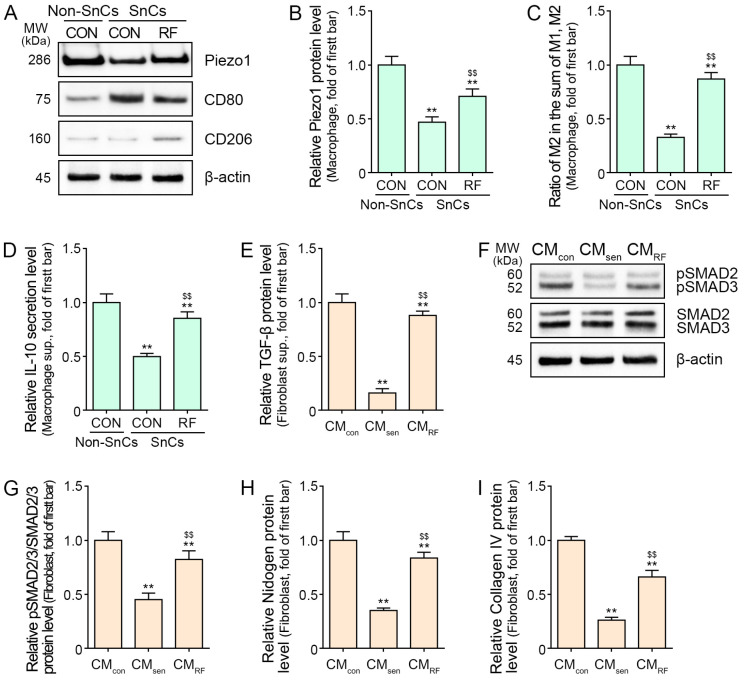
Regulation of M2 polarization and Piezo1, IL-10, TGF-β, SMAD2/3, and DEJ protein levels following RF treatment in senescent cells. (**A**–**C**) Western blot analysis of Piezo1 and CD206 in senescent macrophages following RF treatment. (**D**) ELISA results for IL-10 in senescent macrophages following RF treatment. (**E**) ELISA results for TGF-β in senescent fibroblasts influenced by RF-treated macrophages. (**F**,**G**) Western blot analysis of SMAD2/3 and pSMAD2/3 in senescent fibroblasts influenced by RF-treated macrophages. (**H**,**I**) ELISA results for nidogen and collagen IV in senescent fibroblasts influenced by RF-treated macrophages. Data are presented as mean ± SD of three independent experiments. **, *p* < 0.01 vs. first bar; $$, *p* < 0.01 vs. second bar (Mann–Whitney U test). CM_con_, conditioned media from non-senescent macrophages; CM_RF_, conditioned media from RF-treated senescent macrophages; CM_sen_, conditioned media from senescent macrophages; CON, control; DEJ, dermal–epidermal junction; ELISA, enzyme-linked immunosorbent assay; IL-10, interleukin 10; MW, molecular weight; pSMAD2/3, phosphorylated SMAD2/3; RF, radiofrequency; SD, standard deviation; SnCs, senescent; TGF-β, transforming growth factor beta.

To establish a senescent macrophage model, macrophages were treated with H_2_O_2_ (Figure S2A). The expression levels of *P21* and *P16* were increased by H_2_O_2_ in the macrophage (Figure S2B,C). The expression of Piezo1 was decreased in the senescence macrophage. RF treatment led to an increased expression of Piezo1. CD80 and CD206 are frequently used as M1 and M2 markers, respectively [44]. The proportion of M2 in the sum of M1 and M2 was evaluated for macrophage polarization toward M2. The proportion of M2 was decreased by H_2_O_2_ in the senescent macrophage and increased by RF (Figure 2A–C). IL-10 secretion was decreased in senescent macrophages. RF treatment also resulted in elevated IL-10 secretion (Figure 2D).

To investigate the effect of RF treatment on senescent fibroblasts influenced by RF-treated macrophages, we established an in vitro model with macrophages and fibroblasts (Figure S3). Conditioned media from non-senescent macrophages (CM_con_), senescent macrophages (CM_sen_), or RF-treated senescent macrophages (CM_RF_) were administered to senescent fibroblasts. The expression of *P21* and *P16* were increased by CM_sen_ in the fibroblasts (Figure S4). Treatment with CM_sen_ resulted in decreased TGF-β expression and the expression ratio of phosphorylated SMAD2/3 to total SMAD2/3 (pSMAD2/3/SMAD2/3) in senescent fibroblasts. However, treatment with CM_RF_ resulted in increased TGF-β expression and the expression ratio of pSMAD2/3/SMAD2/3 in senescent fibroblasts (Figure 2E–G). Treatment with CM_sen_ resulted in decreased expression levels of nidogen and collagen IV in senescent fibroblasts. Moreover, treatment with CM_RF_ led to elevated expression levels of nidogen and collagen IV in senescent fibroblasts (Figure 2H,I).

### 2.3. RF Treatment Increases M2 Polarization and HSP47, HSP90, Piezo1, and IL-10 Expression in Aged Skin

**Figure 3:**
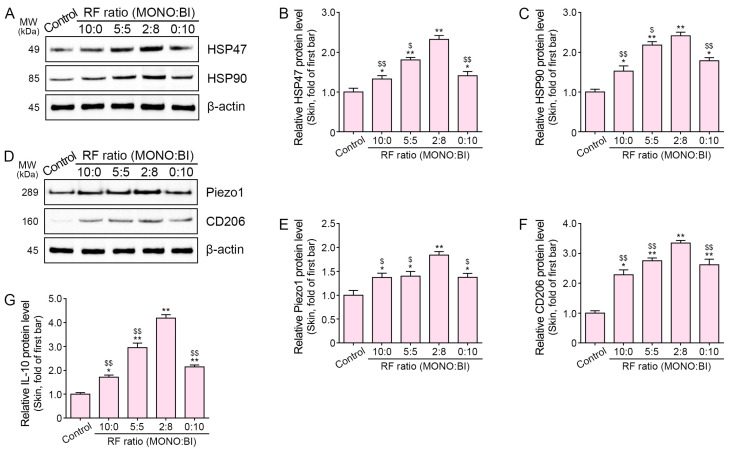
Regulation of HSP47, HSP90, Piezo1, CD206, and IL-10 in aged mice skin following RF treatment. Protein levels were assessed in the skin of 16-month-old (aged) mice following RF treatment. (**A**–**F**) Western blot analysis of HSP47, HSP90, Piezo1, and CD206. (**G**) ELISA results for IL-10. Data are presented as mean ± SD of three independent experiments. *, *p* < 0.05 and **, *p* < 0.01 vs. first bar; $, *p* < 0.05 and $$, *p* < 0.01 vs. fourth bar (Mann–Whitney U test). Moreover, 10:0, 10 monopolar pulses only; 5:5, 5 monopolar pulses followed by 5 bipolar pulses; 2:8, 2 monopolar pulses followed by 8 bipolar pulses; 0:10, 10 bipolar pulses only; BI, bipolar; ELISA, enzyme-linked immunosorbent assay; HSP47, heat shock protein 47; HSP90, heat shock protein 90; IL-10, interleukin 10; MONO, monopolar; MW, molecular weight; RF, radiofrequency; SD, standard deviation.

The expression levels of *P21* and *P16* in aged mice skin were higher than those of young mice skin (Figure S5). Aging mice were subjected to various combinations of monopolar and bipolar RF modes administered in a specific sequence of pulses. These combinations included 10 monopolar pulses only (10:0), 5 monopolar pulses followed by 5 bipolar pulses (5:5), 2 monopolar pulses followed by 8 bipolar pulses (2:8), and 10 bipolar pulses only (0:10) (Figure S6). HSP47 and HSP90 expression was elevated in the aged mice skin 28 days after RF treatment (Figure 3A–C). Furthermore, RF treatment resulted in increased Piezo1, CD206, and IL-10 expression (Figure 3D–G). Notably, the most significant increases were observed with the 2:8 ratio of the monopolar and bipolar RF mode treatment sequence.

### 2.4. RF Treatment Increases TGF-β, SMAD2/3, and DEJ Protein Expression in Aged Mice Skin

**Figure 4:**
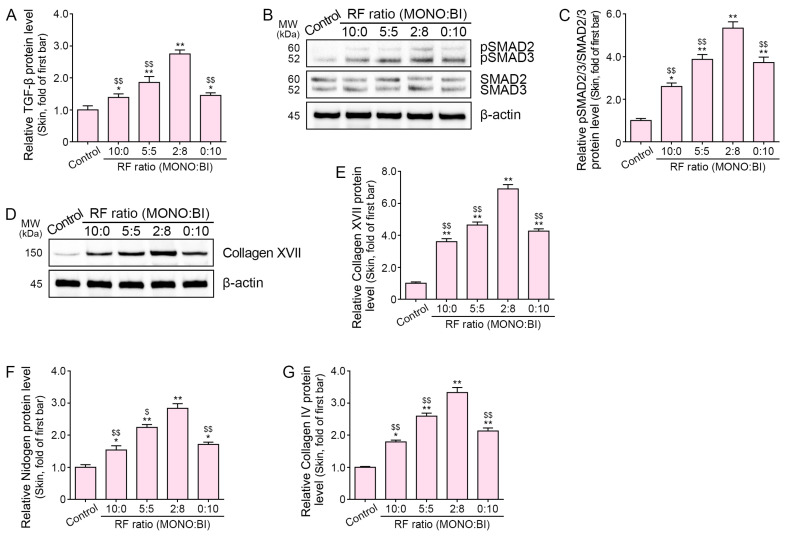
Regulation of TGF-β, SMAD2/3, and DEJ proteins in aged mice skin following RF treatment. Protein levels were assessed in aged mice skin following RF treatment. (**A**) ELISA results for TGF-β. (**B**,**C**) Western blot analysis of SMAD2/3 and pSMAD2/3. (**D**,**E**) Western blot analysis of collagen XVII. (**F**,**G**) ELISA results for nidogen and collagen IV. Data are presented as mean ± SD of three independent experiments. *, *p* < 0.05 and **, *p* < 0.01 vs. first bar; $, *p* < 0.05 and $$, *p* < 0.01 vs. fourth bar (Mann–Whitney U test). Moreover, 10:0, 10 monopolar pulses only; 5:5, 5 monopolar pulses followed by 5 bipolar pulses; 2:8, 2 monopolar pulses followed by 8 bipolar pulses; 0:10, 10 bipolar pulses only; BI, bipolar; ELISA, enzyme-linked immunosorbent assay; IL-10, interleukin 10; MONO, monopolar; MW, molecular weight; pSMAD2/3, phosphorylated SMAD2/3; RF, radiofrequency; SD, standard deviation; TGF-β, transforming growth factor beta.

RF treatment resulted in an increased expression of TGF-β and the pSMAD2/3/SMAD2/3 ratio in aged mice skin (Figure 4A–C). Additionally, RF treatment led to an increased expression of collagen XVII, nidogen, and collagen IV in aged mice skin (Figure 4D–G). These increases were most pronounced when RF was applied using the 2:8 ratio of monopolar and bipolar mode treatment.

### 2.5. RF Treatment Mitigates DEJ Structural Changes

**Figure 5:**
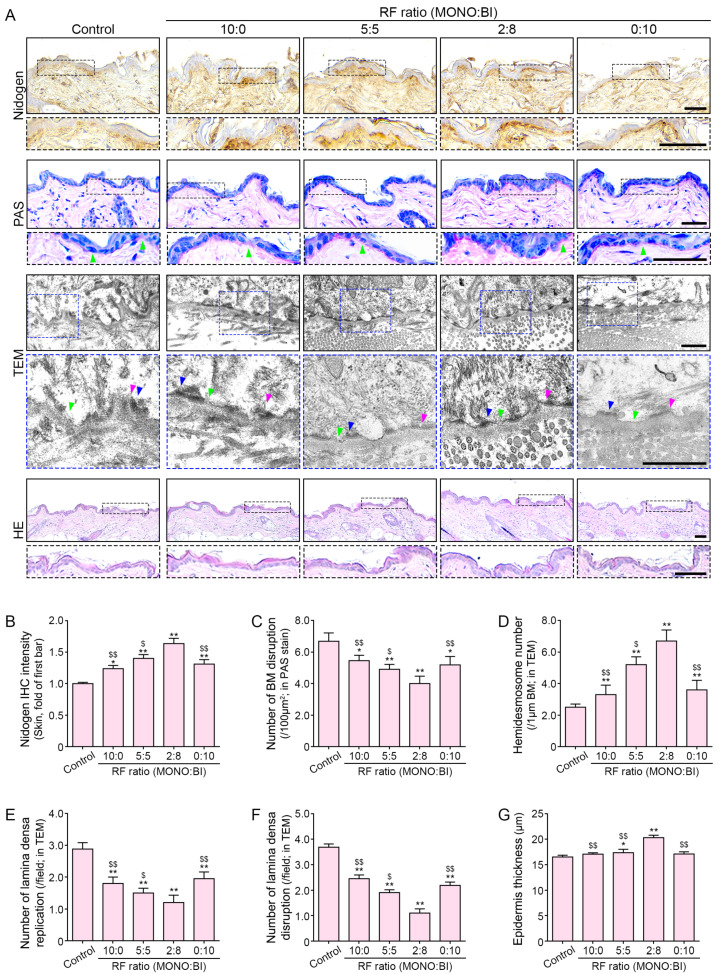
Regulation of DEJ structural changes in aged mice skin following RF treatment. (**A**) Representative images of immunohistochemistry staining for nidogen, PAS staining, TEM, and HE staining in aged mice skin following RF treatment. The green mark represents BM with disruption in PAS staining. Also, in TEM image the green mark represents lamina disruption, magenta represents replication, and blue represents hemidesmosomes. Scale bars = 50 μm, 50 μm, 500 nm, and 100 μm, respectively. (**B**) Quantitative analysis of nidogen protein levels. (**C**) Quantification of BM disruptions (PAS staining). (**D**) Quantification of hemidesmosomes. (**E**) Quantification of lamina densa replications and (**F**) disruptions (TEM). (**G**) Quantitative measurement of epidermal thickness (H&E staining). Data are presented as mean ± SD of three independent experiments. *, *p* < 0.05 and **, *p* < 0.01 vs. first bar; $, *p* < 0.05 and $$, *p* < 0.01, vs. fourth bar (Mann–Whitney U test). Moreover, 10:0, 10 monopolar pulses only; 5:5, 5 monopolar pulses followed by 5 bipolar pulses; 2:8, 2 monopolar pulses followed by 8 bipolar pulses; 0:10, 10 bipolar pulses only; BI, bipolar; BM, basement membrane; DEJ, dermal–epidermal junction; HE, hematoxylin and eosin; IHC, immunohistochemistry; MONO, monopolar; PAS, periodic acid–Schiff; TEM, transmission electron microscopy.

Immunohistochemistry staining for nidogen revealed a notable increase in expression primarily localized to the DEJ following the RF treatment of aged mice skin (Figure 5A,B). This increase was most pronounced when RF was applied using the 2:8 ratio of monopolar and bipolar mode treatment.

Periodic acid–Schiff (PAS) staining demonstrated a reduction in disrupted BM lesions after RF treatment (Figure 5A,C). Transmission electron microscopy (TEM) analysis showed an elevated number of hemidesmosomes in aged mice skin following RF treatment (Figure 5A,D) despite the known age-related increase in the disruption and reduplication of the lamina densa [45]. Remarkably, RF treatment effectively mitigated both lamina densa replication and disruption lesions (Figure 5A,E,F). Additionally, RF treatment led to increased epidermal thickness in the aged mice skin (Figure 5A,G). These changes were most pronounced when RF was applied using the 2:8 ratio of monopolar and bipolar mode treatment.

## 3. Discussion

Extrinsic skin aging, primarily induced by UV exposure, and chronological aging converge to elevate oxidative stress levels in the skin [46,47,48,49,50]. This oxidative burden manifests in diverse forms of skin damage, notably the degradation of collagen fibers and other essential ECM components, facilitated by increased MMP activity [51,52]. Furthermore, increased oxidative stress triggers the upregulation of activator protein 1 (AP-1) and NF-κB, further fueling MMP activation [53]. Additionally, heightened AP-1 expression suppresses ECM protein synthesis by interfering with TGF-β signaling pathways [54,55]. This dual effect—the reduced synthesis and increased degradation of ECM proteins—results in diminished skin resilience and the formation of wrinkles [56].

Extrinsic aging induces notable alterations in the DEJ. In the sun-exposed areas of aged human skin, an increase in lamina densa duplication weakens the connection between the epidermis and dermis [57], a phenomenon mirrored in aged mice [58]. Furthermore, photoaging exacerbates this process, culminating in the destruction of the BM, as evidenced by disruptions observed in lamina densa structures through TEM imaging [59,60].

The BM plays a crucial role in cell survival and proliferation, as well as providing structural support to the DEJ [1,2]. Within the skin epidermis, predominantly composed of keratinocytes, continuous renewal occurs through the proliferation of basal keratinocytes anchored to the BM, with daughter cells migrating toward the upper epidermis [61]. Additionally, the BM orchestrates essential processes including keratinocyte adhesion, differentiation, proliferation, and survival through the regulation of various growth factors and regulatory molecules [62,63]. Therefore, preserving a normal BM function is essential for epidermal regeneration and is acknowledged as a cornerstone of skin rejuvenation.

Piezo1 has emerged as a crucial player in fibrosis-related diseases due to its involvement in promoting SMAD2/3 phosphorylation and TGF-β signaling [64]. Piezo1 activation occurs in response to various mechanical stimuli, including stretching and compression, as well as chemical stimuli such as TGF-β1, resulting in an increase in intracellular Ca^2+^ levels and the subsequent induction of profibrotic responses [65]. Notably, the inhibition of Piezo1 attenuates fibrosis in proximal tubular cells of the kidney (HK2 cells) [65].

Furthermore, mechanical stretching stimuli applied to macrophages induce M2 polarization, ultimately leading to the osteogenic differentiation of stem cells [66]. This mechanical stress-induced polarization of macrophages has been implicated in bone repair by enhancing stem cell survival [67]. The stretching of macrophages triggers the activation of Piezo1 and subsequent elevation of intracellular Ca^2+^, resulting in M2 polarization and increased TGF-β levels [37]. These alterations contribute to the enhanced migration, proliferation, and osteogenic differentiation of bone marrow mesenchymal stem cells [37].

Our study revealed that the RF treatment of senescent macrophages led to an increase in the expression of Piezo1 and the M2 marker CD206. M2 macrophages are pivotal in wound healing as they secrete IL-10, facilitating tissue regeneration without inflammation [68]. Moreover, M2 macrophages secrete various growth factors, such as vascular endothelial growth factor (VEGF) and TGF-β1, which promote vascular formation and tissue regeneration [68,69,70], while stimulating collagen synthesis in fibroblasts [71,72].

RF treatment has emerged as a promising approach to address skin aging by modulating key cellular components such as HSPs and Piezo1. RF treatment induces heat in the skin, stimulating collagen synthesis [26,27,28]. This heat-induced collagen synthesis is accompanied by increased levels of HSPs [28]. Our study aimed to elucidate the potential of RF treatment in enhancing DEJ protein expression and mitigating BM structural degradation.

Our findings demonstrate that RF treatment augmented M2 polarization and IL-10 secretion from senescent macrophages. Additionally, conditioned media from RF-treated senescent macrophages resulted in increased TGF-β production by senescent fibroblasts, accompanied by elevated levels of nidogen and collagen IV in these fibroblasts. Notably, RF treatment also increased the expression of HSP47 and HSP90 in senescent keratinocytes, alongside elevated levels of TGF-β and collagen XVII in these cells. These observations suggest that RF treatment may induce M2 polarization and enhance the secretion of cytokines, such as IL-10, from macrophages, thus activating fibroblasts. Consequently, this stimulation by RF contributes to the heightened expression of DEJ proteins, highlighting its potential to promote skin rejuvenation at the cellular level.

In aged mice skin, RF treatment resulted in an increased expression of HSP47, HSP90, Piezo1, CD206, IL-10, and TGF-β. These changes were accompanied by elevated levels of DEJ proteins such as collagen XVII, collagen IV, and nidogen. Additionally, RF treatment mitigated structural changes such as BM disruption, as evidenced by the reduced disruption of the lamina densa. Moreover, RF treatment increased the number of hemidesmosomes and epidermal thickness in aged mice skin. These effects were most pronounced when RF treatment was applied using the 2:8 sequence of monopolar and bipolar RF modes.

RF devices deliver electrical currents that are converted into heat energy upon contact with tissue [73,74,75,76]. The amount of heat generated varies depending on the electrical current potency and tissue resistance (impedance), with each tissue having a specific impedance [77]. RF modes are divided into two categories based on electrode configuration: monopolar and bipolar modes. In the monopolar mode, an electromagnetic current flows between the electrode and grounding pad, resulting in a volumetric and uniform heat distribution [42,78,79]. Conversely, the bipolar mode typically penetrates tissues less deeply than the monopolar mode but offers a more controlled energy distribution [73]. Heat generation in the bipolar mode occurs solely between two electrodes, resulting in a narrower coverage area compared to the monopolar mode [80,81,82].

Previous research conducted by our group demonstrated that combining sequential monopolar and bipolar modes leads to a greater increase in collagen fiber accumulation compared to using either mode alone [41]. Furthermore, applying the monopolar mode before the bipolar mode results in significantly more collagen compared to the reverse sequence [41]. Although we did not evaluate the exact mechanism by which the order of modes affects collagen fiber accumulation, we speculate that the monopolar mode creates a more favorable tissue environment for modification, thereby enhancing the effectiveness of the bipolar mode [41]. This hypothesis is based on the principle that tissue temperature affects electrical conductivity, with higher temperatures leading to reduced impedance [83]. Consequently, currents are drawn more strongly to areas with lower impedance [76,84,85,86], thereby creating more conducive conditions for the delivery of bipolar currents.

Based on our findings, we hypothesized that the balance between monopolar and bipolar modes in RF treatment could significantly impact its ability to enhance the DEJ. Our results revealed that using the 2:8 sequence of monopolar and bipolar RF modes resulted in the most substantial increase in DEJ protein expression and structural improvement. However, the exact mechanism behind why this sequence outperformed others, such as 5:5, was not evaluated in this study. One possible explanation is that the 2:8 sequence triggered a significant increase in key proteins such as HSP47, HSP90, and Piezo1. Since both HSP expression and Piezo1 expression are influenced by external factors, it is plausible that the 2:8 sequence provided the optimal stimulation to maximize their expression. Nevertheless, further research is needed to fully understand the intricacies of this phenomenon.

## 4. Materials and Methods

### 4.1. RF Treatment System

The RF device used in this study (Sunny; SHENB Co., Ltd., Seoul, Republic of Korea) was equipped with a user interface allowing the adjustment of RF output energy, a selection between 1-MHz and 2-MHz frequencies, the choice of sub-pulses (1–10), and the modification of the polarity sequence (monopolar/bipolar RF) of the sub-pulse. An impedance matching system was incorporated to assess the skin’s impedance after each energy delivery (to compute the correction value) and to uniformly distribute the energy through 25 electrodes for the subsequent energy delivery.

The RF conditions were set to a 2 MHz frequency, a 9.6 W power output, a time of 100 ms, and a total energy of 0.96 J. For cell treatment where it was difficult to use the grounding pad, the experiment was performed in the bipolar RF mode. Aging mice were subjected to various combination sequences of monopolar/bipolar RF modes, with a pulse width of 5 ms and sub-pulse of 10 and delivering energy in the order of monopolar RF followed by bipolar RF. These combinations included 10 monopolar pulses only (10:0), 5 monopolar pulses followed by 5 bipolar pulses (5:5), 2 monopolar pulses followed by 8 bipolar pulses (2:8), and 10 bipolar pulses only (0:10).

A non-invasive tip (Quasar; SHENB Co., Ltd.) was utilized, comprising 25 electrodes designed to control the movement of each electrode. Upon skin contact, each electrode moved up and down, facilitating the adherence of the 25 electrode pins to the skin. The tip was 38.7 mm in length and 29.6 mm in diameter, with 25 electrode pins having a square size of 17.9 mm, an electrode height of 80 mm, and an inter-electrode distance of 2.13 mm. All experiments were conducted using the same tip configuration.

### 4.2. In Vitro Experiments

#### 4.2.1. Cell Culture

Primary human epidermal keratinocytes (HEKn; American Type Culture Collection [ATCC], Manassas, VA, USA) were cultured in growth medium (GM) composed of dermal cell basal medium (ATCC) supplemented with a keratinocyte growth kit (ATCC). Murine macrophages (RAW 264.7 cells, Korea Cell Line Bank, Seoul, Republic of Korea) were cultured in Dulbecco’s modified Eagle medium (HyClone-Cytiva, Marlborough, MA, USA) supplemented with 10% fetal bovine serum (Gibco, Thermo Fisher Scientific, Rockford, IL, USA) and 1% penicillin/streptomycin (Welgene, Gyeongsan, Republic of Korea). Human dermal fibroblasts (CCD-986Sk; ATCC) were cultured in Iscove′s modified Dulbecco′s medium (Welgene) supplemented with 10% fetal bovine serum and 1% penicillin/streptomycin. All cell types were maintained at 37 °C with 5% CO_2_.

#### 4.2.2. Experimental Design

In vitro experiments were conducted to assess the effects of RF treatment on three cell types: keratinocytes, macrophages, and fibroblasts.

To investigate the impact of RF treatment on keratinocytes, cells were initially exposed to 50 μM H_2_O_2_ for 2 h. Subsequently, they were washed with Dulbecco’s phosphate-buffered saline (DPBS; Gibco Thermo Fisher Scientific) and replenished with fresh GM for 72 h. Following this incubation period, RF treatment was administered, and cells were cultured for 48 h. Protein extraction from cell lysates was performed for further analyses (Figure 1A).

For the macrophage senescence induction, a previously established method with slight modifications was employed [87,88,89,90]. Macrophages were treated with 100 μM H_2_O_2_ for 2 h, followed by washing with DPBS and incubation in fresh GM for 72 h. Subsequently, RF treatment was applied, and cells were cultured for an additional 48 h. Supernatant collection was conducted for fibroblast treatment, and protein isolation from cell lysates was carried out for the subsequent experiments (Figure S2A).

The induction of fibroblast senescence involved treatment with 350 μM H_2_O_2_ for 1.5 h, followed by washing with DPBS and a replacement of the medium with fresh GM for 72 h [88]. During the final 24 h of the 72 h GM treatment period, a 1:1 mixture of macrophage supernatant and GM was added, and cells were cultured for an additional 48 h [90]. The macrophage supernatant was used as either CM_sen_ or CM_RF_. Protein extraction from cell lysates was performed for further analyses (Figure S3).

### 4.3. In Vivo Experiments

#### 4.3.1. Mouse Model and Maintenance

C57BL/6 mice (6 weeks old) were obtained from Orient Bio (Seongnam, Republic of Korea). Mice were housed under controlled conditions with a constant temperature of 20–24 °C and 45–55% humidity and bred, and the born pups were initiated when they reached 16 months of age. Throughout the study, mice had ad libitum access to food and water. Ethical approval for this study was obtained from the Gachon University Animal Experiment Ethics Committee (IACUC, approval number LCDI-2023-0150).

#### 4.3.2. Experimental Design

In vivo experiments were conducted to evaluate the efficacy of various sequences of monopolar and bipolar modes in aging mice (Figure S6). Mice were randomly assigned to five groups, and each group had a designated 2 cm × 2 cm area of shaved skin on their backs and then received RF treatments as follows:

(1) Control: gel (SHENB SUNNY COUPLING FLUID; SHENB Co., Ltd.) application only.
(2) RF (10:0): 10 pulses in monopolar mode.
(3) RF (5:5): 5 pulses in monopolar followed by 5 pulses in bipolar mode.
(4) RF (2:8): 2 pulses in monopolar followed by 8 pulses in bipolar mode.
(5) RF (0:10): 10 pulses in bipolar mode.

Following RF treatment, gel (SHENB SUNNY COUPLING FLUID; SHENB Co., Ltd.) was applied to all mice. After 28 days, mice were anesthetized by inhaling 3% isoflurane (HANA Pharm Co., Ltd., Seoul, Republic of Korea) in the presence of 1.5% O_2_. Subsequently, the treated skin was shaved, and skin tissues were collected for further analysis.

### 4.4. Sample Preparation

#### 4.4.1. Protein Isolation

Protein extraction followed the protocol outlined in the EzRIPA buffer kit (ATTO Corporation, Tokyo, Japan). Cells were washed with PBS and then scraped in 600 μL of radioimmunoprecipitation assay (RIPA) buffer. For skin samples, 50 mg of tissue was cut into several pieces and diluted in 600 μL of RIPA buffer. The mixture was homogenized with 10 cycles, each consisting of 40 s of working time followed by 60 s of resting time, and then incubated on ice for 10 min to promote protein solubilization. After homogenization, both cell and tissue samples were sonicated (high power, 10 s of working time followed by 60 s of resting time) and centrifuged at 14,000× *g* for 15 min at 4 °C to isolate the proteins. Protein concentrations were determined using a bicinchoninic acid assay kit (Thermo Fisher Scientific).

#### 4.4.2. Paraffin-Embedded Skin Tissue Block

Skin tissue was fixed in cold 4% paraformaldehyde (Sigma-Aldrich, St. Louis, MO, USA) for 72 h, then placed in a cassette and washed with distilled water. Subsequently, the tissues were processed in a tissue processor (Leica, Wetzlar, Germany), sequentially soaked in 95% and 99% ethanol, followed by xylene (Duksan, Ansan, Republic of Korea), and finally infiltrated with paraffin (Leica). Paraffin-soaked tissue blocks were made in an embedder. Blocks were sectioned into 7 μm thick sections using a microtome (Leica), and sections were placed on coated slides and incubated overnight at 60 °C for attachment.

### 4.5. Western Blot

Cell lysates or skin proteins were mixed with 4× lithium dodecyl sulfate (LDS) sample buffer and 10× sample reducing agent (Thermo Fisher Scientific), followed by heating at 70 °C for 10 min to denature the proteins. Denatured proteins were loaded onto either 3–8% Tris-acetate gels (Invitrogen, Waltham, MA, USA) for 45 min using Tris-acetate buffer (Invitrogen), or 8%, 10%, or 12% gels for 25 min using a MOPS buffer (Invitrogen) at 200 V.

After electrophoresis, the separated proteins were transferred onto polyvinylidene fluoride (PVDF) membranes (Millipore, Burlington, MA, USA) using a semi-dry transfer system (ATTO) at a current of 1 A for 10 min. PVDF membranes were blocked with 5% skim milk with 0.1% Tween 20 (SPL, Pocheon, Republic of Korea) in tris-buffered saline (TTBS) at room temperature for 1 h. Following three washes with 0.1% TTBS, membranes were incubated overnight at 4 °C with primary antibodies (Table S1). After three washes with 0.1% TTBS, membranes were incubated with horseradish peroxidase-conjugated secondary antibodies (1:10,000; Vector Laboratories, Newark, CA, USA) for 1 h at room temperature. Protein bands were visualized using chemiluminescent solutions and imaged with the ChemiDoc Imaging System (Bio-Rad, Hercules, CA, USA). Protein bands were quantified using ImageJ software version 1.53s (the National Institutes of Health; NIH, Maryland, MD, USA), with protein levels normalized to beta-actin bands and compared with the control samples (The first bar in each graph) [91].

### 4.6. Enzyme-Linked Immunosorbent Assay

Microplates were incubated overnight at 4 °C with 0.05 M carbonate and bicarbonate mixed buffer (pH 9.6) and washed three times with 0.1% Tween 20 in phosphate-buffered saline (TPBS). Microplates were then blocked with 5% skim milk in 0.1% TPBS overnight at 4 °C. After three washes with 0.1% TPBS, 50 μg of samples were added to each well and incubated overnight at 4 °C. Primary antibodies (TGF-β, collagen IV, IL-10, and nidogen) diluted in PBS (Table S1) were added to the washed wells, and microplates were incubated overnight at 4 °C. After washing with PBS, a horseradish peroxidase-conjugated secondary antibody (1:1000; Vector Laboratories) was added and incubated at room temperature for 4 h. Tetramethylbenzidinesolution (Sigma-Aldrich) was applied to each well and incubated at room temperature for 10–20 min. Reactions were stopped with 1 N sulfuric acid (Sigma-Aldrich), and measurements were taken using a microplate reader at 450 nm. Protein levels were compared with the control samples (The first bar in each graph).

### 4.7. Staining

#### 4.7.1. Immunohistochemistry

Skin tissue sections underwent deparaffinization and rehydration through sequential transfers through xylene to 100–70% ethanol. After three PBS washes, nonspecific binding was blocked by incubating the slides with normal serum for 1 h at room temperature. Slides were then incubated with primary antibodies overnight at 4 °C (Table S1). Following PBS washing, slides were incubated with biotinylated secondary antibodies (1:200; Vector Laboratories) for 1 h at room temperature. PBS-rinsed slides were incubated with an ABC reagent (Vector Laboratories), washed, and incubated with 3,3′-diaminobenzidine solution (Sigma-Aldrich) for 5 min, resulting in a brown reaction. Counterstaining was achieved by incubating the slides with hematoxylin (KPNT, Cheongju, Republic of Korea) for 30 s, washing with distilled water, dehydrating, and mounting using a DPX mounting solution (Sigma-Aldrich). The stained tissue was scanned using a slide scanner (Motic Scan Infinity 100; Motic, Beijing, China), and intensity measurements were evaluated using ImageJ software version 1.53s (NIH) and presented in comparison with the control (The first bar in each graph) [92].

#### 4.7.2. PAS Staining

Following deparaffinization, skin tissues were immersed in periodic acid (Scytek Laboratories, West Logan, UT, USA) for 5 min and rinsed with distilled water. Subsequently, they were exposed to Schiff’s reagent (Scytek Laboratories) for 15 min and rinsed with running tap water. Samples were then treated with hematoxylin (Scytek Laboratories) for 2 min and rinsed with distilled water. Processed samples were dehydrated, mounted using a DPX mounting solution (Sigma-Aldrich), and observed the slide scanner (Motic). All images were analyzed for BM disruption using ImageJ software version 1.53s (NIH).

#### 4.7.3. Hematoxylin and Eosin Staining

Skin tissues were deparaffinized and rehydrated. Hematoxylin (DAKO, Glostrup, Denmark) was applied for 30 s followed by eosin (Sigma-Aldrich) for 30 s. Slides were rinsed with distilled water, dehydrated, and mounted with a DPX mounting solution (Sigma-Aldrich). Stained slides were observed the slide scanner (Motic). All images were analyzed for epidermal thickness using ImageJ version 1.53s software (NIH).

### 4.8. Transmission Electron Microscopy

Skin tissue samples measuring 1 mm × 1 mm were fixed in 2% glutaraldehyde/2% paraformaldehyde in 0.1 M phosphate buffer (pH 7.4) for 24 h. After washing with 0.1 M phosphate buffer, the sections underwent additional fixation in 1% OsO_4_ in 0.1 M phosphate buffer for 2 h, followed by dehydration in an ethanol series (50, 60, 70, 80, 90, 95, and 100%; 10 min each). Dehydrated sections were permeated with propylene oxide for 10 min, embedded using the Poly/Bed 812 Embedding Kit (Polysciences, Inc., Warrington, PA, USA) for 12 h, and polymerized in an electron microscopy oven at 65 °C for 12 h. Subsequently, 200 nm sections were obtained using a diamond knife on an ultramicrotome and stained with toluidine blue for light microscopy.

Further processing involved thinning the sections to 80 nm using an ultramicrotome, placing them on a copper grid, and staining with 3% uranyl acetate for 30 min, followed by double staining with 3% lead citrate for 7 min.

Imaging was conducted at an acceleration voltage of 80 kV using a transmission electron microscope (JEM-1011, JEOL, Tokyo, Japan) equipped with a MegaView III CCD camera (Soft Imaging System, Münster, Germany). The analysis of hemidesmosome and lamina densa replication, as well as lamina densa disruption, was performed using TEM images within ImageJ version 1.53s software (NIH).

### 4.9. Quantitative Reverse Transcription–Polymerase Chain Reaction (qRT-PCR)

The qRT-PCR and melting curve analysis were performed using a QuantStudio^TM^ 3 real-time PCR (Thermo Fisher Scientific). mRNA levels were normalized by ACTB and compared to control (The first bar in each graph) [93] (Table S2).

### 4.10. Statistical Analysis

Group comparisons were conducted using the Kruskal–Wallis test, followed by the Mann–Whitney U test for post hoc comparisons. The results are presented as the mean ± standard deviation (SD). Statistical analyses were performed using SPSS version 26 (IBM, Armonk, NY, USA).

## 5. Conclusions

In conclusion, our study highlights the potential of RF treatment as a promising intervention against skin aging, elucidating its ability to modulate key cellular components, including HSPs and Piezo1. Notably, RF treatment induced a significant upregulation of HSP47, HSP90, Piezo1, IL-10, and TGF-β expression in aged mice skin, indicating its involvement in cellular stress responses and anti-inflammatory pathways.

Moreover, RF treatment effectively enhanced the expression of critical proteins at the DEJ, such as collagen XVII, nidogen, and collagen IV, thereby enhancing the DEJ’s structural integrity. Significantly, RF treatment mitigated aging-related disruptions of the DEJ structure by reducing lamina densa disruption and promoting hemidesmosome formation, suggesting its potential to ameliorate age-associated skin abnormalities. These findings collectively underscore RF treatment’s promise for skin rejuvenation, addressing both the cellular and structural aspects of aging skin.

## Data Availability

All data are contained within the article.

## Supplementary Materials

The following supporting information can be downloaded at: https://www.mdpi.com/article/10.3390/ijms25105178/s1.
