# Supplementary material for: Radiofrequency Treatment Attenuates Age-Related Changes in Dermal–Epidermal Junctions of Animal Skin
Source: Int J Mol Sci. 2024 May 9;25(10):5178. doi: 10.3390/ijms25105178 (PMC11120932; doi:10.3390/ijms25105178)
Supplement: Supplementary file 1 [file ijms-25-05178-s001.zip › ijms-2984840-supplementary.pdf]

International Journal of *Molecular Sciences*

MDPI

Article

Radiofrequency Treatment Attenuates Age-Related Changes in Dermal–Epidermal Junctions of Animal Skin

Kyung-A Byun [1,2,3,†], Hyoung Moon Kim [4,†], Seyeon Oh [3], Sosorburam Batsukh [1,3], Kuk Hui Son [5,*] and Kyunghee Byun [1,3,6,*]

1 Department of Anatomy & Cell Biology, College of Medicine, Gachon University, Incheon 21936, Republic of Korea; kabyun95@gachon.ac.kr (K.-A.B.); sosorburam72@gachon.ac.kr (S.B.)
2 LIBON Inc., Incheon 22006, Republic of Korea
3 Functional Cellular Networks Laboratory, Lee Gil Ya Cancer and Diabetes Institute, Gachon University, Incheon 21999, Republic of Korea; seyeon8965@gmail.com
4 Maylin Anti-Aging Center Ilsan, Goyang 10391, Republic of Korea; drmac12@me.com
5 Department of Thoracic and Cardiovascular Surgery, Gachon University Gil Medical Center, Gachon University, Incheon 21565, Republic of Korea
6 Department of Health Sciences and Technology, Gachon Advanced Institute for Health & Sciences and Technology (GAIHST), Gachon University, Incheon 21999, Republic of Korea
* Correspondence: dr632@gachon.ac.kr (K.H.S.); khbyun1@gachon.ac.kr (K.B.); Tel.: +82-32-460-3666 (K.H.S.); +82-32-899-6511 (K.B.)
† These authors contributed equally to this work.

Citation: Byun, K.-A.; Kim, H.M.; Oh, S.; Batsukh, S.; Son, K.H.; Byun, K. Radiofrequency Treatment Attenuates Age-Related Changes in Dermal–Epidermal Junctions of Animal Skin. *Int. J. Mol. Sci.* **2024**, *25*, 5178. https://doi.org/10.3390/ijms25105178

Academic Editor: Terrence Piva

Received: 16 April 2024
Revised: 3 May 2024
Accepted: 7 May 2024
Published: 9 May 2024

Copyright: © 2024 by the authors. Submitted for possible open access publication under the terms and conditions of the Creative Commons Attribution (CC BY) license (https://creativecommons.org/licenses/by/4.0/).

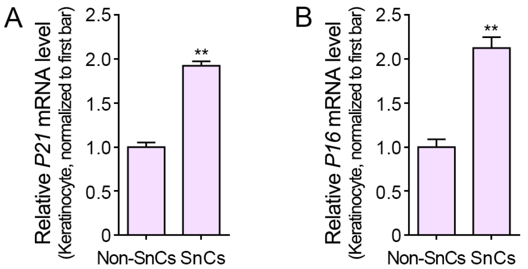

Figure S1. Expression of *P21* and *P16* of senescence marker in non-senescent and senescent keratinocytes. (**A**,**B**) PCR analysis of *P21* and *P16* in keratinocytes. Data are presented as mean ± SD of three independent experiments. **, $p < 0.01$ vs first bar. CON, control; PCR, polymerase chain reaction; SnCs, senescent; SD, standard deviation.

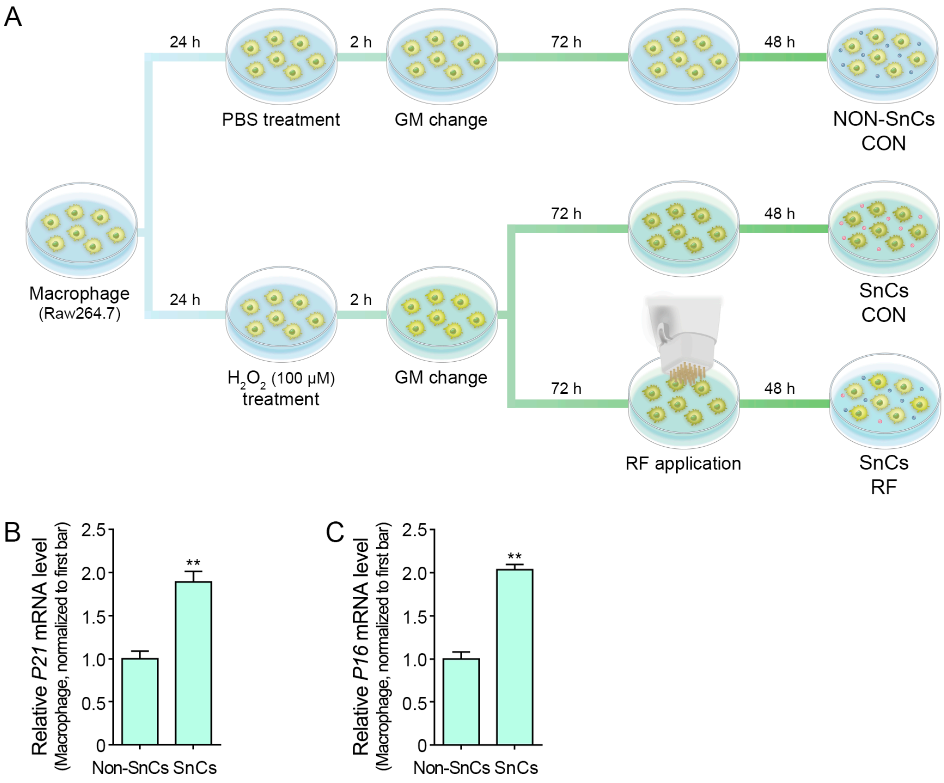

Figure S2. Schematic illustrating H_2O_2-induced senescence in macrophages for evaluation of RF treatment and expression of *P21* and *P16*. (**A**) Schematic representation of RF treatments administered to senescent macrophages. (**B**,**C**) PCR analysis of *P21* and *P16* in macrophages. Data are presented as mean ± SD of three independent experiments. **, $p < 0.01$ vs first bar. CON, control; GM, growth medium; PBS, phosphate-buffered saline; PCR, polymerase chain reaction; RF, radiofrequency; SnCs, senescent; SD, standard deviation.

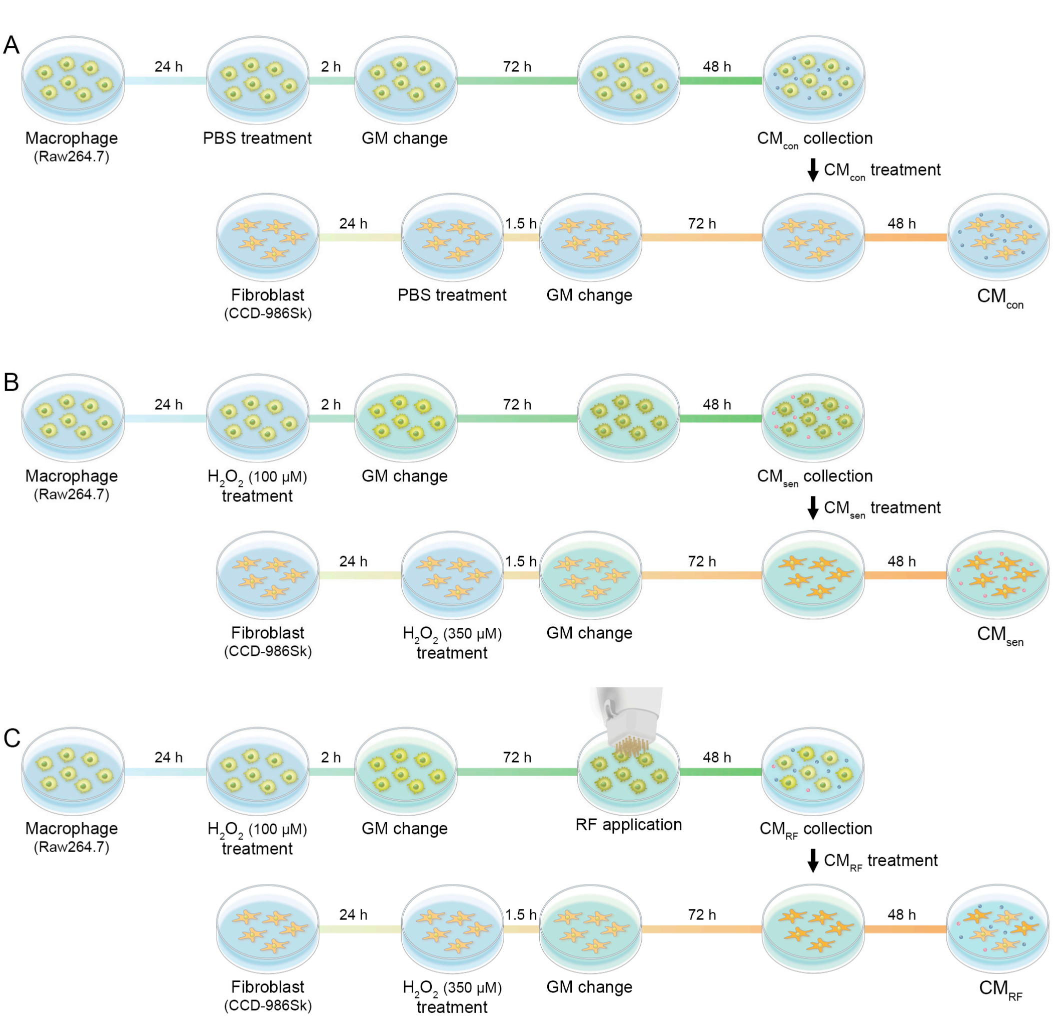

Figure S3. Schematic illustrating H_2O_2-induced senescence in fibroblasts for evaluation of RF treatment. (**A**) Schematic representation of non-senescent fibroblasts influenced by non-senescent macrophages. (**B**) Schematic representation of senescent fibroblasts influenced by senescent macrophages. (**C**) Schematic representation of senescent fibroblasts influenced by RF-treated senescent macrophages. CM_{con}, conditioned media from non-senescent macrophages; CM_{RF}, conditioned media from RF-treated senescent macrophages; CM_{sen}, conditioned media from senescent macrophages; GM, growth medium; PBS, phosphate-buffered saline; RF, radiofrequency.

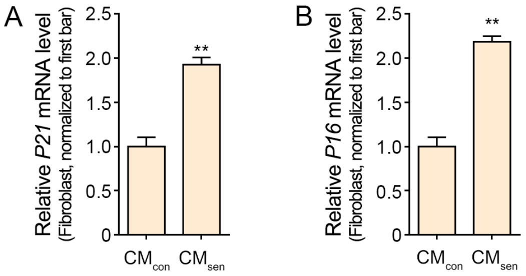

Figure S4. Expression of *P21* and *P16* of senescence marker in non-senescent and senescent fibroblasts. (**A**,**B**) PCR analysis of *P21* and *P16* in fibroblasts. Data are presented as mean ± SD of three independent experiments. **, $p < 0.01$ vs first bar. CM_{con}, conditioned media from non-senescent macrophages; CM_{sen}, conditioned media from senescent macrophages; PCR, polymerase chain reaction; SnCs, senescent; SD, standard deviation.

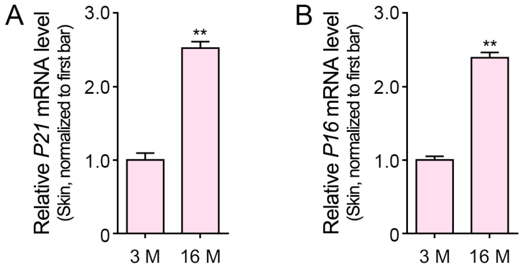

Figure S5. Expression of *P21* and *P16* of senescence marker in young and aged mice skin. (**A**,**B**) PCR analysis of *P21* and *P16* in 3-month-old mice and 16-month-old mice skins. Data are presented as mean ± SD of three independent experiments. **, $p < 0.01$ vs first bar. 3 M, 3-month-old mice; 16 M, 3-month-old mice; PCR, polymerase chain reaction; SD, standard deviation.

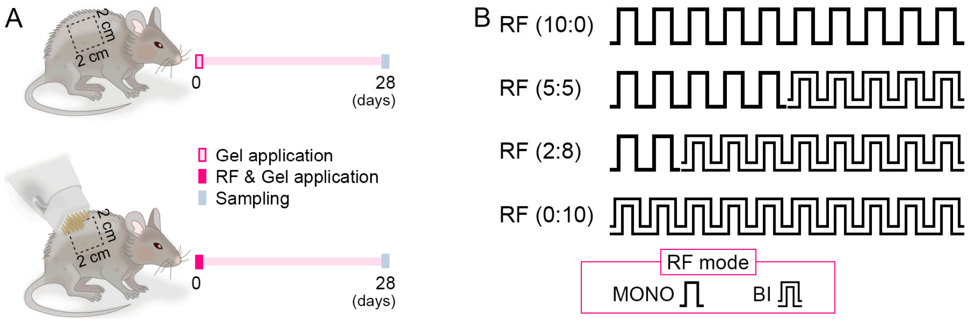

Figure S6. Schematic illustrating the experimental procedure of RF treatment in aged mice. (**A**) Schematic representation of RF treatment of 16-month-old mice. (**B**) Schematic representation of RF ratio. 10:0, 10 monopolar pulses only; 5:5, 5 monopolar pulses followed by 5 bipolar pulses; 2:8, 2 monopolar pulses followed by 8 bipolar pulses; 0:10, 10 bipolar pulses only; BI, bipolar; MONO, monopolar; RF, radiofrequency.

Table S1. List of antibodies for Western blot (WB), Enzyme-linked immunosorbent assay (ELISA), and Immunohistochemistry (IHC).

Antibody	Company	Catalog No.	Dilution rate		
			WB	ELISA	IHC
HSP47	Abcam	Ab77609	1:1,000		
HSP90	Gene Tex	GTX109753	1:5,000		
TGF-β	Abcam	Ab64715		1:5,000	
Collagen XVII	Affinity	DF2913	1:5,000		
Collagen IV	Invitrogen	PA1-28534		1:5,000	
Piezo1	Protein tech	15939-1-AP	1:500		
CD206	Santa cruz	Sc-58986	1:1,000		
IL-10	Fine Test	FNab04211		1:5,000	
pSMAD2/3	Cell signaling	8828	1:1,000		
SMAD2/3	Cell signaling	8685	1:1,000		
Nidogen	Santa cruz	sc-47773		1:5,000	1:100

Table S2. List of primers for quantitative reverse–transcription polymerase chain reaction.

Gene (Organism)	Primer sequences	
ACTB (human)	Forward	5'-CTC GCC TTT GCC GAT CC-3′
	Reverse	5'-TCT CCA TGT CGT CCC AGT TG-3′
P21 (human)	Forward	5'-GGA GAC TCT CAG GGT CGA AAA-3′
	Reverse	5'-GCT TCC TCT TGG AGA AGA TCA G-3′
P16 (human)	Forward	5'-CAC TTT CCT GGG CAA CAA ATA-3′
	Reverse	5'-CTT GCG GTC ATC ATC GTA GTT-3′
Actb (mouse)	Forward	5'-AAA GCC TAT TTC TGC CAG GAC-3′
	Reverse	5'-TCA TAG GTC ACG TAG CCC ACT-3′
P21 (mouse)	Forward	5'-ACC AGA GGC AGT AAC CAT GC-3′
	Reverse	5'-TGC TTC TAC AAA CCC ACA AAT G-3′
P16 (mouse)	Forward	5'-GGT TCT TGG TCA CTG TGA GGA T-3′
	Reverse	5'-GCA CGA ACT TCA CCA AGA AAA-3′
